# Assessment of recipients’ characteristics, transfusion appropriateness, and utilization pattern of blood and blood products in Jimma Medical Center, Jimma, Ethiopia

**DOI:** 10.1371/journal.pone.0250623

**Published:** 2021-04-26

**Authors:** Tufa Feyisa, Girum Tesfaye Kiya, Wondimagegn Adissu Maleko

**Affiliations:** 1 Hematology Laboratory Unit of Jimma Medical Center, Jimma University, Jimma, Ethiopia; 2 School of Medical Laboratory Sciences, Jimma University, Jimma, Ethiopia; 3 Clinical Trial Unit, Jimma University, Jimma, Ethiopia; University of Mississippi Medical Center, UNITED STATES

## Abstract

**Background:**

As blood transfusion remains life-saving and is being frequently prescribed, a greater number of its practice is unnecessary or inappropriate. This important clinical intervention is reported as one of the five overused medical treatments, with gross over-ordering and whole blood transfusions as the sole component being common in developing countries. Study of recipient’s demographics, clinical conditions, appropriate blood utilization, and continuous clinical audits for quality assurance and service improvement plan are important factors to this practice. This study was designed to assess the recipient’s characteristics, blood type distributions, appropriateness of blood transfusion, and utilization practice of the big medical center.

**Methods:**

Institution based cross-sectional study was conducted from February 1 to June 30, 2018. Data were collected using a structured data collection format prepared for this study. All transfusion prescriptions were followed from requisition up to completion. Patient’s age, sex, requesting departments, hemodynamics, number and component of units requested and issued, and units transfused were collected. Transfusion appropriateness was assessed by a criterion-based method while blood utilization was calculated.

**Results:**

A total of 545 units of blood for 425 patients were cross-matched of the 809 units of total blood prescribed. The mean and median age of transfused individuals was found to be 27.47 ±15.28 years and 26 years respectively, and 65.4% females most in reproductive age groups. O and A Rhesus-positive blood types were the two major blood groups observed. Overall 82.1% of transfusions were appropriate; while only 27.8% of patients received appropriate components as 96.5% of individuals received a whole blood transfusion. Significant blood utilization was recorded with a C/T ratio of 1.05, TP% of 100%, and TI of 1.23.

**Conclusion:**

Much of the transfusion recipients were relatively young aged and females, most in the reproductive age group. Although whole blood was used as a sole component, significant blood transfusion utilization and appropriateness were recorded; while appropriate component transfusion was recorded to be significantly low. Local transfusion guidelines and appropriate component preparation and utilization are required to improve the sub-optimal blood component transfusion practice.

## Introduction

It has been realized by humans that blood is essential, *“vital force and essence of life”* since the beginning of ancient period, similar to the study and interest in knowledge of blood [1–3]. However, until the description of the circulatory system and 20^th^-century laboratory investigation-oriented practice dated back from 1900–01, the benefit of blood infusion did not emerge [3–6]. Since then, transfusion has played a significant role in medical and surgical practices [1, 6–10]. Nowadays, it is estimated that about 10% of hospital admissions needed blood transfusion [11]. Thus, this practice is becoming a common procedure [11–14] as blood and blood products being prescribed by physicians frequently [15] for transfusion. And also, it has been reported that blood transfusion is among the five overused medical treatments throughout the world [13, 15, 16].

Currently, allogeneic blood transfusion remains a common practice in the management of life-saving clinical situations like trauma, surgical blood loss, and in cases of severe anemia. For these reasons, however, the provision of hospital transfusion services remains under greater pressure in the context of blood safety, availability, and cost [17] depending on the overall healthcare status of that specific area [2, 10]. Blood transfusion therapy aims in providing the patient with appropriate blood components for specific hematologic deficiencies that the patient has rather than transfusing whole blood [18]. This practice is supported by the central dogma of transfusion *“giving a patient only the blood components that the patient needs”* [19, 20]. Thus, an adequate supply of safe blood and blood products and accessibility of that blood to transfusion needs, and appropriate clinical use of it are the two key factors for the effectiveness of transfusion [2, 21, 22].

According to the World Health Organization’s (WHO) report, there is a remarkable difference in the use of blood transfusion in different hospitals, clinical specialties, and between different teams in a specific area, which leads to the inappropriate use of blood and blood products [22]. To benefit from transfusions therapeutic value, it is important to study recipients’ demographics, such as age and sex and clinical conditions like ward and transfusion inducing clinical findings in forecasting the demand for blood transfusion in particular settings [6, 23], and to devise blood transfusion needs, particularly in resource-limited areas where there is scarce blood supply [23, 24]. It is always wise to consider that blood is an expensive and scarce resource [11], so that appropriate clinical use of blood and blood products, avoiding unnecessary transfusions, and use of simple transfusion alternatives are some of the strategies of WHO blood transfusion service (WHO/BTS) [25] advocated. Therefore, blood should be appropriately transfused to treat a certain condition in clinical practice [21] by ensuring that *“Is blood transfusion necessary in this patient*? If so, *“ensure right blood*, *for the right patient*, *at the right time*, *at the right place”* [8].

A review document reported by Giuliano G. indicated that appropriateness of transfusion practices should systematically mean that *“blood components are transfused only when there is evidence for potential benefit*, *there are no valid alternatives*, *safe and quality products are available*, *and risks and benefits are carefully assessed before the decision to transfuse are made”* [26]. Besides, overall transfusion safety is not only on the side of correct blood collection, product preparation, and administration but also on the ability of correct interpretation of this clinical intervention needed [27]. With this regard, researchers suggested that 5 to 58% of blood transfusions carried out were estimated to be *“unnecessary or inappropriate”* [27, 28].

Several study reports indicated that a large number of surgical blood transfusion over-orderings, and blood utilization practices ranging from only 5% to 40% [29]. Likewise, estimates in developing countries also indicated that 40% to 70% of transfused patients showed gross over-ordering [30]. In South Africa, 7–10% of annual blood wastage was reported because of over-ordering [31], and the blood utilization rate in India was found to be 28% [32], in Kuwait, 13.6% [33], in Ethiopia 43.6% [34], and in Nigeria 69.7% [35]. Even in trauma patients, less than 50% of blood utilization was reported in 2004 by *Olatunji et al*. [36].

Boral Henry in 1975 was the first to use the determination of cross-match-to-transfusion (C/T or C:T) ratio for evaluation of blood utilization and reservation [34, 37]. This C/T ratio has to be within the index value of 2:1 or 2.5:1 [30, 34, 37], although WHO lets this range as 3:1 to be ideal for surgical patients [22]. The probability of blood transfusion (TP%) with a considered value of >30% to be is expected desirable. Meaning that at least 30% of cross-matched blood needed a transfusion, with any figure less than this number being said to be unjustifiable [30, 37] or in surgical procedures blood usage, less than 30% would be included under the category of group and screen [22].

Another threat to the judicious use of this scarce resource is failing to use components of blood instead of whole blood. Although people usually donate whole blood, it is rarely given as a transfusion in modern medical practices [38]. Blood transfusion history in Africa is assumed to be introduced beyond the “life-saving” role [17, 39–42] of the procedure. It is mainly associated with the acceptance and spread of modern technology of medicine, reported as started in 1921 [7] and 1940 according to WHO’s report, which became more important during the 1980s following the emergence of HIV/AIDS [7, 43, 44]. The Ethiopian Red Cross Society (ERCS) was the pioneer for the establishment and organization of blood transfusion services in Ethiopia and founded the first blood transfusion center in 1969 in Addis Ababa. The service later expanded as a network covering some major hospitals in the country [45, 46] and reverted to government-owned activity in 2010 after it is handed over to the Federal Ministry of Health (FMoH). Currently, there are 25 blood banks strategically positioned across all regions of the country to ensure access to safe and adequate blood supply, based on the nationally proposed regional blood bank services organogram [46].

Globally, of 80 million (in 2011) [47] to 118.5 million approximate units (in 2020) [48] of blood donated each year, only 40% collected in high-income countries, however only 2 million units in sub-Saharan Africa, in contrary; demand for blood transfusions is excessive in the later region due to maternal morbidity, malnutrition, and a heavy burden of infectious diseases such as malaria [22, 49]. Generally, little attention has been given to the clinical safety component of the blood thereby minimizing the risks involved with blood transfusion practices and improving the blood supply system and appropriate clinical use of blood within the health care facilities. Although there are limited studies that indicate the current approach of blood transfusion practices and the need assessment for blood components therapy in Ethiopia, this study aimed at identifying the demographic characteristics of recipients, blood type distribution, and the practice of appropriate clinical utilization patterns of blood and blood products in the study area.

## Materials and methods

### Study setting

A facility-based cross-sectional study was conducted in Jimma medical center (JMC), Jimma University, southwest Ethiopia. Blood transfusion requests of 425 patients from different clinical departments were included consecutively. The study was conducted from February 1 to June 30, 2018. Any blood-prescribing physician and patient for whom a blood transfusion intervention was clinically indicated and blood or blood component requested during the study period were included voluntarily. Currently, the medical center was developing local blood and blood product transfusion SOP, maximum surgical blood ordering schedule (MSBOS), and transfusion guideline through the newly established transfusion committee.

### Data collection and analysis

Socio-demographic and blood transfusion-related data were collected using a checklist developed after reviewing different works of literature. The checklist included age, gender, clinical wards, pre-transfusion hemoglobin level, blood group of patient and unit, transfusion indication, number of units requested, blood components indicated and administered, prescribing physician, ethical issues/consent/ for blood transfusion. The data were extracted from manually completed blood bank request papers and followed prospectively to the wards for transfusion completion by a trained medical laboratory technologist. Completeness and consistency check was done before data were entered, coded, organized, and analyzed by SPSS® software version 23.0 (*Armonk*, *NY*: *IBM Corps*.); and C/T ratios, TI and TP% were calculated by Microsoft® excel (Microsoft® office 2010, Microsoft Corporation, USA).

### Statistics

Appropriate descriptive and frequency summaries, percentages, and ratios were carried out to describe the study variables. Results of the study were presented using narration statements, tables, and figures.

Appropriateness of blood transfusion was decided based on the algorithm-based criteria set below [50–52].

#### Transfusion appropriateness algorism

- *Whole blood transfusion*: *inappropriate in most cases except in massive hemorrhage and exchange transfusion*
- *Packed RBC*: *Hemoglobin trigger value of 7 g/dl*
  - *If the patient hemoglobin is <5 g/dl* *packed RBC only*
  - *Acute anemia*: *If patient hemoglobin 7–8*.*9 g/dl*, *and hemodynamically unstable*
  - *≥9g/dl* *and difficult in controlling hemorrhage*
  - *Chronic anemia*: *<8g/dl*, *with signs and symptoms of anemia*
  - *Pregnant mothers*: *<5 with symptoms of anemia and 5–7 g/dl associated with co-existing diseases*
  - *Pediatrics*: *<4 g/dl transfuse*, *4–5 g/dl with signs of respiratory distress and cardiac failure*
  - *Neonates*: *<13 g/dl with severe pulmonary disease or CHF*, *acute blood loss shock*
  - *Neonates 8–10 g/dl* *stable but with signs and symptoms of anemia*
  - *Neonates <7* *stable and without signs and symptoms of anemia*
  - *Adult medicine*: *by considering blood transfusion in the case of hypoxia*
  - *Surgery*: *≥10 g/dl no need of transfusion*, *7–8 g/dl in a well-compensated healthy individual not necessary*, *but if the surgical patient is with uncompensated or inadequately compensated anemia*, *major surgery with anticipated significant blood loss with the procedure transfuse packed RBC*.

#### The following mathematical steps were used for calculations of C/T ratio, TP%, TI, and I:T: [34, 37, 53]

- *Cross-match to transfusion ratio (C/T ratio)*: *number of units cross-matched/number of units transfused*. *A ratio of ≤2*.*5 is considered indicative of efficient blood use*.
- *Transfusion probability (TP %)*: *number of patients transfused over the number of patients cross-matched by 100*. *A value of ≥30% considered being significant blood usage*.
- *Transfusion index (TI)*: *number of units transfused/ number of patients cross-matched*. *A value of ≥0*.*5 is considered significant blood utilization*.
- *Issue to transfusion index (I*:*T)*: *number of blood issued to patients over the actual number of units transfused to patients*.

### Quality assurance

The checklist was prepared in the English language. Orientation was given to all data collectors regarding the procedures to be followed during the data collection, then collected data were reviewed and checked for completeness and consistency daily by the PI, and two supervisors.

### Ethics approval and consent to participate

Ethical clearance was obtained from the Ethical Review Board of Institute of Health, Jimma University. Permission from the Medical director of JMC and written informed consent from each participant was taken prior to any data collection and follow up. Written informed consent was also taken from parents and legal guardians in cases of minors for the use of their clinical and demographic data for this study. Transfusion was solely initiated by authorized physician as part of regular patient management, where all transfusion practices were followed after that till completion, and no transfusion was initiated by investigators. Confidentiality was maintained at all levels of the study and all the information obtained was used for this study only.

## Results

### Demographic and clinical data

A total of 425 blood transfusion requests for the same number of patients were made and followed in this study. Most blood transfused patients (65.4%; 278/425) denoted were females, with the majority of them were in the reproductive age group (5–49 years). The median age of patients in this study was 26 years (interquartile range of 15), more than half (57.5%) of total patients in the age group of 25–64 years, only less than 15% of blood transfused patients under the age of 15, and fewer than 4% were at least 65 years of age. Blood group O Rhesus-positive and A Rhesus-positive were found to be the two commonly observed once with 38.8% and 32.2% of total patients, respectively. Almost half (48.2%) of the total transfusion requests made and blood transfusions were from the internal medicine department. Hemorrhage due to trauma, pregnancy, and delivery-related transfusions, surgery, and other systemic disease-related internal hemolysis accompanied with different degrees of anemia were the major reasons for the indication of blood transfusion. Most of the study participants, 306/425 (72.0%) had severe anemia; Hb value of <80 g/L (<8 g/dL), and 96.7% of the total patients had co-morbidity associated with the reason to transfusion (Table 1).

**Table 1:** Demographic and clinical data of blood transfusion recipients according to their sex at JMC, southwest Ethiopia, February 1 to June 30, 2018 (n = 425).

		Total recipient		Male		Female	
		*n	*(%)	**n	**(%)	**n	**(%)
**Age group**	0–14 years	66	15.5	41	62.1	25	37.9
	15–24 years	101	23.8	32	31.7	69	68.3
	25–64 years	244	57.4	67	27.5	177	72.5
	≥ 65 years	14	3.3	7	50.0	7	50.0
**Blood group**	A+	137	32.2	43	31.4	94	68.6
	A-	17	4.0	3	17.6	14	82.4
	B+	69	16.2	27	39.1	42	60.9
	B-	4	0.9	4	100.0	0	0.0
	AB+	23	5.4	6	26.1	17	73.9
	AB-	0	0.0	0	0.0	0	0.0
	O+	165	38.8	61	37	104	63.0
	O-	10	2.4	3	30	7	70.0
**Ward of admission**	Medical	205	48.2	69	33.7	136	66.3
	Surgical	95	22.4	44	46.3	51	53.7
	***GynObs and Maternity	70	16.5	0	0.0	70	100
	Pediatrics	51	12.0	31	60.8	20	39.2
	***EM-OPD	4	0.9	3	75	1	25
****Anemia class and hemoglobin range	Mild anemia (110-129g/L)	0	0.0	0	0.0	0	0.0
	Moderate anemia (80–109 g/L)	119	28.0	46	38.7	73	61.3
	Severe anemia (<80 g/L)	306	72.0	101	33.0	205	67.0
**Comorbidity**	Pre-eclampsia	15	3.5	0	0.0	15	100.0
	Renal failure	34	8.0	13	38.2	21	61.8
	Cardiorespiratory disease	52	12.2	14	26.9	38	73.1
	Chronic lung disease	26	6.1	11	42.3	15	57.7
	Acute infection	105	24.7	37	35.2	68	64.8
	Diabetes	44	10.4	10	22.7	34	77.3
	Trauma	32	7.5	8	25.0	24	75.0
	RVI infection	101	23.8	43	42.6	58	57.4
	No	16	3.8	11	68.8	5	31.2
**Transfusion indications**	Acute blood loss	78	18.4	31	39.7	47	60.3
	Anemia	327	76.9	110	33.6	217	66.4
	Hematologic malignancy	13	3.1	4	30.8	9	69.2
	Exchange transfusion	5	1.2	2	40.0	3	60
	Hemolytic disease of the fetus and new born (HDFN)	2	0.5	0	0.0	2	100.0
**TOTAL**		**425**	**100.0**	**147**	**34.6**	**278**	**65.4**

*n(%) = of the total population,

**n(%) = of the group.

***GynObs = Gynecology and Obstetrics, EM-OPD = emergency outpatients department.

****Anemia = based on WHO anemia classification [54].

### Blood utilization and comparison of blood and blood product request and administration

A total of 809 units of blood and blood product requests were made during the study period. Of this, 40.7% (173/425) and 33.6% (143/425) of request papers contain one and two units of blood and blood products per request paper respectively, with only 2/425 papers had five units of blood products request. The mean ±SD and range of units ordered per requisition observed were 1.90 ±0.91, range of 4 (1–5 units per request); while only 67.1% of the total requested units were issued for transfusion with 77.2% (328/425) blood issuance was with one unit of blood product per requisition.

About 56% (236/425) of blood transfusion indications issued as per physicians prescription, whereas the rest not issued due to patient stability (57.1%), low product stock in the bank (41.3%), and postponement of the scheduled procedure (1.6%).

Most blood transfusion requests were for whole blood transfusions, similarly, 96.6% of blood product issuance was made as whole blood, with only 1.9% as a concentrated red cell (CRBC), 0.7% platelet concentrate, 0.5% FFP, and no granulocyte concentrate issued. Although whole blood was issued as the sole component for transfusion, 95.3% (405/425) issued prescriptions have been utilized. Low blood in stock, patients’ stability, and change in procedure schedule were still the major reasons for the return of issued blood to the blood bank (Table 2).

**Table 2:** Indicators of blood transfusion practice at JMC, southwest Ethiopia, February 1 to June 30, 2018 (n = 425).

Variables		n (%)
**Number of units indicated/prescribed for transfusion**	1 unit	173 (40.7)
	2 units	143 (33.6)
	3 units	88 (20.7)
	4 units	19 (4.5)
	5 units	2 (0.5)
**Number of units issued for transfusion**	1 unit	328 (77.2)
	2 units	79 (18.6)
	3 units	15 (3.5)
	4 units	3 (0.7)
	5 units	0 (0.0)
**Components indicated/prescribed for transfusion**	Whole blood	302 (71.1)
	*CRBC	113 (26.6)
	*FFP	3 (0.7)
	Platelet concentrate	6 (1.4)
	Granulocyte concentrate	1 (0.2)
**Components administered for transfusion**	Whole blood	412 (96.9)
	CRBC	8 (1.9)
	FFP	2 (0.5)
	Platelet concentrate	3 (0.7)
	Granulocyte concentrate	0 (0.0)

*CRBC = concentrated red blood cell; FFP = fresh frozen plasma.

The recipient’s and transfused unit blood type relationship showed that 410/425 (96.5%) of patients were transfused with group-specific blood or blood components. On the other hand, O Rhesus-positive and O Rhesus-negative blood type units were commonly used as second choice blood for transfusion in cases of group-specific blood or blood product stock-out (Table 3).

**Table 3:** Blood type distribution of recipient according to blood type transfused JMC, southwest Ethiopia, February 1 to June 30, 2018 (n = 425).

		Blood group of a unit (n /%)							
		A+	A-	B+	B-	AB+	AB-	O+	O-
**Blood group of a recipient**	**A+**	**131(30.8)**	-	-	-	-	**-**	**6 (1.4)**	-
	**A-**	-	**13 (3.1)**	-	-	-	-	-	**4 (0.9)**
	**B+**	-	-	**65 (15.3)**	-	-	**-**	**4 (0.9)**	-
	**B-**	-	-	-	**4 (0.9)**	-	-	-	-
	**AB+**	**1 (0.2)**	-	-	-	**21 (4.9)**	**-**	**1 (0.2)**	-
	**AB-**	**-**	-	-	-	**-**	**-**	**-**	-
	**O+**	-	-	-	-	-	**-**	**165 (38.8)**	-
	**O-**	-	-	-	-	-	-	-	**10 (2.4)**
**Total**		**132(31.1)**	**13 (3.1)**	**65 (15.3)**	**4 (0.9)**	**21(4.9)**	**0 (0.0)**	**176 (41.4)**	**14 (3.3)**

Blood component stratification showed that whole blood was utilized more in patients of the Internal Medicine department (n = 201; 48.8%) followed by Surgery, Gynecology and Obstetrics, and Maternity. Anemia was recorded as the leading cause of transfusion induction caused by underlying diseases, acute bleeding due to surgery, delivery, and trauma. Whole blood was utilized in 319 (77.4%) anemic patients, while only 6 of anemic patients received CRBC (Table 4).

**Table 4:** Utilization of blood components across different clinical departments, JMC, southwest Ethiopia, February 1 to June 30, 2018.

		Blood component			
		Whole blood	CRBC	FFP	Platelet concentrate
Ward of Admission	Medical	201 (48.8)	3 (37.5)	0 (0.0)	1 (33.3)
	Surgical	92 (22.3)	2 (25.5)	0 (0.0)	1 (33.3)
	*GynObs and Maternity	69 (16.7)	1 (12.5)	0 (0.0)	0 (0.0)
	Pediatrics	47 (11.4)	1 (12.5)	2 (100.0)	1 (33.3)
	*EM-OPD	3 (0.7)	1 (12.5)	0 (0.0)	0 (0.0)
Transfusion inducing broad diagnosis	**Anemia	108 (26.2)	3 (37.5)	0 (0.0)	0 (0.0)
	Pancytopenia	19 (4.6)	2 (25.5)	1 (50.0)	1 (33.3)
	Malignancy	83 (20.1)	1 (12.5)	0 (0.0)	2 (66.7)
	Hemorrhage and Hemolysis	178 (43.2)	2 (25.5)	1 (50.0)	0 (0.0)
	Surgery	24 (5.8)	0 (0.0)	0 (0.0)	0 (0.0)
Actual transfusion indications	Acute blood loss	75 (18.2)	2 (25.0)	0 (0.0)	1 (33.3)
	**Anemia	319 (77.4)	6 (75.0)	1 (50.0)	1 (33.3)
	Hematologic malignancy	12 (2.9)	0 (0.0)	1 (33.3)	0
	Exchange transfusion	4 (1.0)	0 (0.0)	0 (0.0)	1 (33.3)
	Hemolytic disease of the fetus and newborn	2 (0.5)	0 (0.0)	0 (0.0)	0 (0.0)

*GynObs = Gynecology and Obstetrics, EM-OPD = emergency outpatients department.

**Anemia = based on WHO anemia classification [54].

### Appropriate utilization of blood transfusion

**Fig 1:**
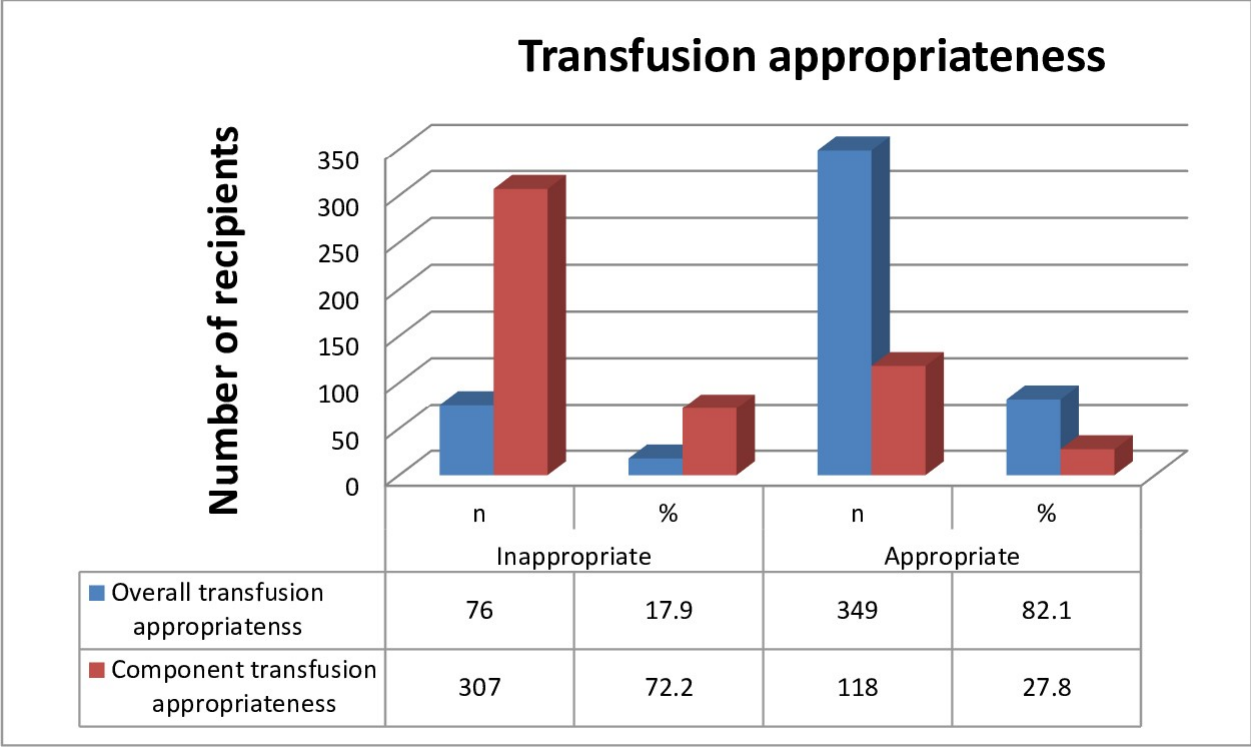
Blood transfusion practice appropriateness distribution at JMC, southwest Ethiopia, February 1 to June 30, 2018 (n = 425).

The overall prevalence of blood transfusion appropriateness in this study was 82.1%, however, only 27.8% of transfused patients received appropriate blood components (Fig 1). Most appropriate transfusions were observed in age groups of 15–24 and 25–64 years of age. On the other hand, female recipients experienced higher appropriate blood transfusions; however, 41.2% of patients with moderate anemia were inappropriately transfused with blood or blood product. Hemodynamically unstable patients received maximum blood transfusion more accurately (92.6%) than others and patients who were comorbid with acute infections, cardiorespiratory disease, and pre-eclampsia showed a relatively higher degree of inappropriate blood transfusions. Emergency OPD and surgery departments showed a higher degree of appropriate blood or blood product transfusions; although the number of transfusions in emergency OPD was very low. (Table 5).

**Table 5:** Blood transfusion practice appropriateness distribution according to demography and clinical variables at JMC, southwest Ethiopia, February 1 to June 30, 2018 (n = 425).

Variables		Overall appropriateness of transfusion					
		Recipients		Inappropriate		Appropriate	
		n	%	n	%	n	%
Age group	0–14 years	66	15.5	15	22.7	51	77.3
	15–24 years	101	23.8	14	13.9	87	86.1
	25–64 years	244	57.4	44	18.0	200	82.0
	≥65 years	14	3.3	3	21.4	11	78.6
Sex	Male	147	34.6	33	22.4	114	77.6
	Female	278	65.4	43	15.5	235	84.5
**Hemoglobin range and anemia class	Mild 110–129 g/L	0	0.0	0	0.0	0	0.0
	Moderate 80–109 g/L	119	28.0	49	41.2	70	58.8
	Severe <80 g/L	306	72.0	27	8.8	279	91.2
Hemodynamics	Unstable	338	79.5	25	7.4	313	92.6
	Stable	79	18.6	48	60.8	31	39.2
	Unidentified	8	1.9	3	37.5	5	62.5
Comorbidity	Pre-eclampsia	15	3.5	3	20.0	12	80.0
	Renal failure	34	8.0	6	17.6	28	82.4
	Cardiorespiratory disease	52	12.2	11	21.2	41	78.8
	Chronic lung disease	26	6.1	3	11.5	23	88.5
	Acute infection	105	24.7	26	24.8	79	75.2
	Diabetes	44	10.4	7	15.9	37	84.1
	Trauma	32	7.5	3	9.4	29	90.6
	RVI infection	101	23.8	15	14.9	86	85.1
	No	16	3.6	2	12.5	14	87.5
Ward of admission	Medicine	205	48.2	41	20.0	164	80.0
	Surgery	95	22.4	12	12.6	83	87.4
	*GynObs Maternity	70	16.5	12	17.1	58	82.9
	Pediatrics	51	12.0	11	21.6	40	78.4
	*EM-OPD	4	0.9	0	0.0	4	100.0
Actual transfusion indications	Acute blood loss	78	18.4	18	23.1	60	76.9
	Anemia	327	76.9	55	16.8	272	83.2
	Hematologic malignancy	13	3.1	2	15.4	11	84.6
	Exchange transfusion	5	1.2	1	20.0	4	80.0
	Hemolytic disease of the fetus and newborn	2	0.5	0	0.0	2	100.0
Time of transfusion	Day	357	84.0	47	13.2	310	86.8
	Night	68	16.0	29	42.6	39	57.4

*GynObs = Gynecology and Obstetrics, EM-OPD = emergency out patients department.

**Anemia = based on WHO anemia classification [54].

A total of 545 units of blood and blood components were cross-matched for transfusion, as all the study participants included in this study were received at least one unit of blood or blood component (TP of 100%). A higher number of crossmatch was observed in patients with the age group of 25–64 years (n = 315 units; 57.8%) followed by patients of age group 15–24 years (n = 127 units; 23.3%). Overall age groups mean ±SD of C/T ratio and TI were 1.09 ±0.13 and 1.20 ±0.13 respectively. A higher proportion occupying two age groups had the same C/T ratio of 1.03 and TI of 1.22 and 1.25. Generally, 271 (49.7%), 117 (21.5%), and 91 (16.7%) units of blood and blood product were cross-matched for Internal Medicine, Surgery, and GynObs/Maternity departments; of which 263 (97.1%), 108 (92.3%) and 88 (96.7%) of units of blood or blood products were transfused in respective departments, with mean ±SD of C/T ratio of 1.04 ±0.03 and TI of 1.17 ±0.11. As anemia was a common problem that could lead to blood transfusions; just as 73.9% (n = 403) of blood cross matches were performed for severely anemic patients; with Hb value of 80 g/L (<8g/dL). The majority of cross matches (n = 469; 86.1% units) and blood transfusion practices (n = 449; 86.1% units) were recorded during the daytime of clinical services. Overall cross-match to transfusion ratio (C/T ratio) was almost 1 (1.05 observed); with transfusion probability (TP %) of 100% and transfusion index (TI) of 1.23. These findings of C/T ratio throughout all patients for different clinical variables were <1.5 with observed TI of >0.5 indicated that the overall blood transfusion utilization in the medical center was significant (Table 6).

**Table 6:** Blood transfusion prescription and dispatch distribution to clinical variables at JMC, southwest Ethiopia, February 1 to June 30, 2018 (n = 425).

Variables	Recipient		Cross-matched units (n)		Issued units (n)	Transfused units (n)		C/T ratio	TP%	TI
	n	%								
**Age**										
0–14	66	15.5	78		78	74		1.05	100	1.15
15-24yrs	101	23.8	127		127	123		1.03	100	1.22
25-64yrs	244	57.4	315		315	305		1.03	100	1.25
≥65 yrs	14	3.3	25		23	19		1.32	100	1.36
**Gender**										
Male	147	34.6	183		183	175		1.05	100	1.19
Female	278	65.4	362		360	346		1.05	100	1.24
**Ward**										
Medical	205	48.2	271		269	263		1.03	100	1.26
Surgical	95	22.4	117		117	108		1.08	100	1.14
*GynObs and Maternity	70	16.5	91		91	88		1.03	100	1.26
Pediatrics	51	12.0	62		62	58		1.07	100	1.20
*EM-OPD	4	0.9	4		4	4		1.00	100	1.00
**Anemia class										
Mild 11–12.9	0	0.0	0		0	0		-	-	-
Moderate 8–10.9	119	28.0	142		142	137		1.04	100	1.16
Severe <8	306	72.0	403		401	384		1.05	100	1.25
**Time of transfusion**										
Day	357	84.0	469		467	449		1.04	100	1.26
Night	68	16.0	76		76	72		1.06	100	1.06
**Overall blood transfusion prescription and dispatch**										
Blood transfusion indicators				Observed value			Overall blood utilization status			
Cross match to transfusion ratio (C/T ratio)				545/521 = 1.05			Significant blood utilization			
Transfusion probability (TP %)				(425/425)*100 = 100%			Significant blood utilization			
Transfusion index (TI)				521/425 = 1.23			Significant blood utilization			

*GynObs = Gynecology and Obstetrics, EM-OPD = emergency out patients department.

**Anemia = based on WHO anemia classification [54].

## Discussion

Blood transfusion in clinical practice has significant importance in medical and surgical practices with a life-saving role [7, 23]. The therapeutic benefit of this practice needs to be supported by evidence through continuous review and evaluations of demographic characteristics, clinical conditions of transfusion recipients, and appropriate clinical utilization of blood and blood products [6, 23]. Blood is essentially a scarce resource and also transfusion poses a potential danger to recipients [7, 9, 11, 15]; thus, these two vital points in transfusion medicine requires appropriate measures need to be considered in potential avoiding of unnecessary blood transfusions by following appropriate patient blood management, exerting efforts in avoiding transfusion errors, and use of simple transfusion alternatives [25, 55].

Much of the transfusion recipients in this study were found to be females (65%) and younger cohorts (mean ±SD age of 27.47 ±15.28 years; median age of 26) of different clinical departments. Subsequently, similar studies in sub-Saharan Africa countries reported comparable results: 69.1% in Nigeria [23], 62.3 in Zimbabwe [56], Uganda female to male ratio (F:M) of 1.3 [57], 57.7% in Malawi [58], 58% in sub-Saharan Africa hospital [59], 55.2% in Kenya [60]. The low median age in this study indicates the structural distribution of the general population in sub-Saharan Africa regions which is mainly characterized by a higher proportion of younger people, as opposed to the aging population in developed countries [56]. Blood transfusion practice in this region is also higher because of the higher prevalence of malaria and its endemicity, higher prevalence of multifactorial severe anemia, pregnancy-related hemorrhages, and effects of some complicated labour during delivery [23, 43, 44, 56, 60].

The distribution of ABO and Rhesus blood group observation in this study was consistent with that of reported blood donors of Jimma [61], Ethiopia [62–64], Nigeria [23] as type O, and A Rhesus-positive frequency are predominate groups, while AB Rh-negative and O Rh-Negative frequency being significantly low in the general population as well as in transfused patients. *Golassa et al*. in 2017 reported a 19.37% Rh D-negative proportion being substantial as compared to the national and global data reports, where Rh D-negative distribution in Ethiopia is assumed to be very low, and the phenotype distribution variation is significantly different between populations [64]. On the other hand, AB blood group distribution was reported as the least blood group in Jimma town by *Bekele et al* in 2016 and only 4.3% of blood donors were AB blood types as reported by *Alemu et al* in 2016 [61]. This knowledge of blood type distribution among transfused patients is important for planning and efficient utilization of blood and avoiding critical blood shortages [23], ensuring patients receive group-specific blood or blood components during transfusion, avoids unnecessary wastage of unused blood, and facilitates the use of component preparation from this rare blood types for long storage and use.

Although blood component preparation and use have been increasingly advocated for sub-Saharan Africa countries, whole blood was found to be used as a sole component for transfusion, as the former is a resource-demanding practice [65]. The majority of patients (97%) in this study received whole blood contrary to the rare or no use of this component for transfusion in recent medical practices [38, 65]. Anemic individuals can be treated effectively by transfusing concentrated red cells, as this product contains minimal plasma content [60]. This finding is higher than reports of Nigeria (71.6%) [23], National Referral Hospitals in sub-Saharan Africa (60.2%) [59], Kenya (60.2%) [60], and Zimbabwe (8.4%) [56] of use of whole blood component for transfusions. WHO’s blood safety status survey 2006 [43] reported that 24/39 African countries reported use of more than 75% of whole blood transfusions, whereas the 2010 survey [44] indicated 60.3% of whole blood collections separated into components, and also 2016 Global Status Report on blood safety and availability recorded that whole blood transfusions in low-income countries by World Bank income group was 36.9–98.7% (median 85%) [66]. This is because of the limited capabilities for component productions, equipment shortage, and maintenance problems of blood banks and hospitals in the region, and the common practice of requesting whole blood due to the unavailability of blood components for transfusion [20, 23, 24, 50] as well as the use of whole blood and packed RBC transfusions in the treatment of pregnancy-related bleeding and anemias and for pediatric transfusions [65].

This study also recorded that majority of recipients were from medical (48.2%) and surgical (22.4%) departments. Comparable results were reported in Kenya [60], Nigeria [23], Malawi (medical ward) [58], and Uganda (medical ward) [59], as most patients received transfusion therapy from medical and surgical wards. This could be due to high anemia prevalence, higher HIV and other infectious diseases burden, and surgical procedure hemorrhages resulting in the need for blood and blood product transfusions [60]. All transfusion recipients were diagnosed with anemia, 72% of them having a severe type. This makes anemia to be the major reason for the indication of transfusion in medical and surgical wards [56, 60]. *Mafirakureva et al’s*. report of Zimbabwe indicated that the majority of patients were transfused due to pregnancy and childbirth at maternity and delivery wards, although the reason for transfusion was still anemia [56] and *Kipkulei et al*. from Kenya also reported 62.8% anemia among transfused individuals. Besides being the most common cause of transfusion indication [60, 65], the majority of anemic and transfused patients in this study also had underlying acute or chronic infections (54.6%: acute infections, HIV, and lung disease), which could result in anemia of chronic diseases, the common type of anemia among hospitalized patients [60, 67, 68].

Overall, 82.1% transfusion appropriateness was recorded in this study which is lower than a report of 90.0% by *Tiwari et al*. [18] but higher than the study reported by *Richa et al*. of 62.6% [25] and *Zhu et al*. of 37.3% [69], but only 27.8% of recipients received appropriate component transfusions. This was due to the use of whole blood as a sole component for transfusion in resource-limited areas [20, 50] and also 97% whole blood transfusion in this study of all patients. In developed countries, whole blood is seldom used as a component for blood transfusion rather than its therapeutic products [26, 27]. Although blood components were used for transfusion, studies reported a higher incidence of clinical inappropriateness of blood component transfusions at different parts of the world [25, 27, 28]. On *Richa et al*.*’s* report [25] 44.5% FFP, 39.8 CRBC, 27.7% PLT, and 19.7% whole blood transfusions, on *Zhu et al*.*’s* report [69] 56.3% plasma, 30.9% CRBC, 25.2% cryoprecipitate and 14.1% PLT transfusions, on *Diaz et al*.’s report [27] 78.6% CRBC transfusions, and on *Shandler et al*.*’s* report [55] 59.3% CRBC transfusions were inappropriate. The mean number of units prescribed and transfused per patient in this study indicated 1.90 ±0.91 and 1.28 ±0.56. Comparable mean units of blood prescription and transfusion per patient findings were reported by *Mahfouz et al*. from Egypt [14], *Tegu et al*., and *Zewdie et al*. from Ethiopia [53, 70]. *Natukunda et al*. in Uganda [57]; but a lower report of 1.09 units of blood per patient prescription was also reported by *Belayneh et al*. in Gondar [34]. This lower mean prescribed units of blood per patient was because of only surgical patients involved in the Gondar study as compared to all patients from different departments followed in this study. Higher prevalence of anemia (esp. severe type anemia), absence of maximum surgical ordering schedule in the medical center, and anemia associated with acute and chronic underlying diseases might be the cause for higher units of blood prescriptions per request paper.

The overall incidence of blood utilization in this study was 95.6%, C/T ratio of 1.05, 100% TP, and TI of 1.23. This significant blood utilization is in concordance with varied study reports of sub-Saharan Africa; C/T ratio of 1.3 in Uganda [57], 1.03–1.07 C/T ratios in South Africa [28], 1.17 C/T ratio in Addis Ababa only for severely anemic individuals [53]; 2.3 C/T ratio, 47% TP, 0.77 TI in Gondar [34]; 1.52 C/T ratio, 65.4% TP, 0.65 TI in Pakistan [71]; and C/T ratio of 2±1 in Spain but significantly better off than other study reports [27]; 7.6 C/T ratio, 15.3% TP, 0.29 TI and 4.9 C/T ratio, 22.7% TP, 0.44 TI in Addis Ababa [53, 70]; >2.5 C/T in 98% of patients with only in 21.1% of them having TI of >0.5 (30); 2–12 C/T ratio, 12.5–100% TP, only 0.25–0.75 TI in Bir hospital, Nepal [33]; 38.7% utilization, 3.5 C/T ratio, 0.5 TI in Egypt [14]; 3.71 C/T ratio (7.81 if all requisitions were cross matched), 16.83% TP, 0.31 TI in Iran [37]. This discrepancy of significant blood utilization observed was because the majority of study reports made by following primarily surgical patients; however, this study followed all patients from different departments and higher prevalence of anemia; 98.4% (especially severe anemia), which may lead to ordering and transfusion of much blood and blood product units per patient.

## Conclusion

This study finding demonstrated that much of the blood transfusion recipients were female patients, most of them in the reproductive age group. The majority of young-aged recipients are indicators of the high burden of anemia, acute and chronic infections causing anemia of chronic diseases, malaria endemicity, and other associated factors like pregnancy and childbirth, trauma, and accidents and surgery. Most transfusions made in the medical ward were due to anemia and associated comorbidities. Blood type O Rh-positive and A Rh-positive are the two most common characteristics in the population which can be used for appropriate blood collection planning and utilization. This study showed significant blood utilization with high overall transfusion appropriateness, although very low transfusion of appropriate blood components might serve as an indicator of diminished component preparation and utilization capability in the blood bank. Besides these, the absence of local blood transfusion prescription and utilization guidelines, MSBOS, and SOP contributed to the suboptimal utilization and transfusion appropriateness recorded. Blood and blood product was prescribed to patients any time when needed, and the blood bank provides available blood from the bank, evidenced by the suboptimal utilization of the medical practice. Therefore, we recommend the medical center develop its local blood transfusion guideline and SOP, MSBOS for efficient utilization of blood, and the blood bank to develop the capacity of blood component preparation and distribution.

## Abbreviations

C/T: Crossmatch to transfusion ratio
TI: Transfusion index
TP%: Transfusion probability
WHO: World Health Organization
BTS: Blood Transfusion Service
ERCS: Ethiopian Red Cross Society
HIV: Human Immunodeficiency Virus
AIDS: Acquired Immunodeficiency syndrome
FMoH: Federal Ministry of Health
JMC: Jimma Medical Center
